# Realization of multiple orbital angular momentum modes simultaneously through four-dimensional antenna arrays

**DOI:** 10.1038/s41598-017-18264-3

**Published:** 2018-01-09

**Authors:** Chao Sun, Shiwen Yang, Yikai Chen, Jixin Guo, Shiwei Qu

**Affiliations:** School of Electronic Engineering, University of Electronic Science and Technology of China (UESTC), Chengdu, 611731 P. R. China

## Abstract

Electromagnetic waves carrying orbital angular momentum (OAM) in radio frequency range have drawn great attention owing to its potential applications in increasing communication capacity. In this paper, both single-pole single-throw (SPST) switches and single-pole double-throw (SPDT) switches are designed and implemented. Optimal time sequence allows four-dimensional (4-D) circular antenna array to generate multiple OAM-carrying waves as well as enhance the field intensity of each OAM-carrying wave. A novel experimental platform is developed to measure the phase distribution when the transmitting antenna and the receiving antenna operate at different frequencies. The good agreement between the measurement and simulation results demonstrate that 4-D circular antenna array is able to generate multiple OAM modes simultaneously. Furthermore, the superiority of the 4-D circular antenna array in receiving and demodulating multiple OAM-carrying signals is validated through the filter and bit error rate (BER) simulations.

## Introduction

Electromagnetic (EM) waves carry both linear momentum and angular momentum. Angular momentum can be divided into spin angular momentum (SAM) and orbital angular momentum (OAM). The former is associated with the polarization, whereas the latter leads to helical phase fronts. OAM was firstly discovered in optical regime in the 1990s^1^. An OAM-carrying beam has a phase front of exp (*jℓθ*) that “twists” in a helical fashion as it propagates, where *ℓ* is the OAM order (*l* = 0, ±1, ±2, …) and *θ* is the azimuthal angle. Due to the unlimited number of orthogonal eigenstates (*l*), OAM provides another degree of freedom to manipulate the optical field. OAM therefore has the potential to tremendously increase the spectral efficiency and data capacity of optical communication systems^2^. Moreover, OAM in optical communication system potentially provides improved security performance^3^. Very recently, OAM was proposed in radio frequency range instead of being only realized in optical area. In 2007, the first radio frequency OAM simulation was performed^4^. After that, OAM-carrying waves received great attention in wireless communications. Inspired from works in optics, variable spiral phase plate (SPP) structures are designed in different approaches, including flat plate with index-varying permittivity^5^, split-ring frequency selective surface (FSS) spiral phase plate^6^, spiral reflector^7^ and twisted parabolic reflector^8^. Another solution to generate OAM-carrying waves is through using uniform circular antenna arrays (UCA) with phase shifting between successive elements. It is generally composed of *N* short dipoles, where each dipole is fed by the same signal with a ±2πℓ/*N* phase difference with its neighboring element^9^. Because of the finite number of antenna elements in an UCA, the maximum OAM mode number is limited to |*l*| < *N*/2^2^. This is the current state-of-art approach for generating OAM-carrying waves. However, the above antennas/antenna arrays suffer from the same limitation that they cannot produce multiple OAM modes at an instant. While for OAM communications, simultaneous multiple modes are beneficial for increasing the capacity. To overcome this limitation, time modulated circular antenna array (TMCA), which belongs to the class of four-dimensional (4-D) antenna arrays, was proposed to generate multiple OAM-carrying waves at several harmonic frequencies simultaneously^10^. Characterized by the use of time as an additional degree of freedom, 4-D antenna arrays have lots of advantages in antenna array design and applications, such as ultra-low sidelobe level (SLL) arrays^11^, harmonic beam-forming^12,13^, signal transmission^14,15^, radar systems^16^, secure communication^17^, cognitive radio systems^18^ and sidelobe blanking radar system^19^. However, the capability of 4-D antenna array in OAM-carrying wave generations has not been experimentally validated. Moreover, the performance improvement in system level of the OAM generated by 4-D antenna arrays still remained unexplored.

In this work, a novel experimental platform for transmitting and receiving antennas operating at different frequencies is developed, aiming to observe the attractive feature of 4-D circular antenna array in generating multiple OAM–carrying waves. Optimized time sequences for both SPST switches and SPDT switches are presented to increase the radiation power and radiation efficiency of OAM modes that are generated by 4-D antenna array. In addition, system level advantages of the 4-D antenna array technique are demonstrated. The capability of 4-D circular antenna array in receiving and demodulating OAM-carrying BPSK signals are investigated through the filtered waveforms and BER performance.

## Results

### Theory basic

For simplicity, consider an *N*-element 4-D circular antenna array with its elements placed equidistantly along a circle with a radius of *R* (see Supplementary Note 1 for the configuration of the 4-D circular antenna array). Each antenna element is connected to a high speed RF switch, which is controlled by the circuit board programmed for specific time sequences. Suppose that the center frequency is *f*_0_ and the time-modulation frequency is *f*_*p*_, which implies the time-modulation period *T*_*p*_ = 1/*f*_*p*_. Pulse shifting approach is adopted, and the periodic on-off time function for the *k*th element by using SPST RF switch is given by

1 $${U}_{k}(t)=\{\begin{array}{c}1,{t}_{k}\le t\le {t}_{k}+{\tau }_{k}\\ 0,\quad \quad \quad others\end{array},0\le {t}_{k}\le 1,0\le {\tau }_{k}\le 1$$

where *t*_*k*_ and *τ*_*k*_ denote the normalized switch-on time instant and normalized switch-on time duration for the *k*th element, respectively. Decompose Equation (1) into Fourier series in frequency domain, the equivalent complex excitation for the *k*th element at the *m-*th harmonic frequency can be written as

2 $$\begin{array}{rcl}{a}_{mk} & = & \frac{1}{{T}_{p}}{\int }_{0}^{{T}_{p}}{U}_{k}(t){e}^{-j2\pi {f}_{p}t}\cdot dt={\tau }_{k}{\rm{sinc}}(\pi m{\tau }_{k}){e}^{-j\pi m(2{t}_{k}+{\tau }_{k})}\\  & = & [{\tau }_{k}{\rm{sinc}}(\pi m{\tau }_{k}){e}^{-j\pi m{\tau }_{k}}]{e}^{-jm(2\pi {t}_{k})}\end{array}$$

where *a*_*mk*_ will equal to the rotating factor of the OAM-carrying waves in UCA by carefully design the time sequence. *τ*_*k*_ (*k* = 1, 2 …, *N*) is expected to be the same to ensure the amplitude excitation of each element is the same. *t*_*k*_ is supposed to be *t*_*k*_ = (*k* – 1)/*N* (k = 1, 2 …, *N*) in order to realize a ±2π*m*/*N* phase difference as compared with its neighbor elements.

In practice, 4-D antenna array based on SPST switches usually have very high reduction in radiation power of each OAM-carrying wave, due to the power absorption of the off-state absorptive switches. Thus, SPDT switch is introduced to solve this problem. The phase difference between the two states (“1” state and “−1” state) of SPDT switch is 180°. Since the switches are theoretically always in the “ON” states, there’s no power loss in the absorptive switches of the feed network. The periodic time function for the *k*th element by using SPDT switch is given by

3 $${U}_{k}(t)=\{\begin{array}{c}1,{t}_{k}\le t\le {t}_{k}+{\tau }_{k}\\ -1,\quad \quad \quad others\end{array},0\le {t}_{k}\le 1,0\le {\tau }_{k}\le 1$$

where *t*_*k*_ and *τ*_*k*_ denote the normalized time instant and normalized time duration of the switch connecting to “1” state for the *k*th element, respectively. Decompose Equation (3) into Fourier series in frequency domain, the equivalent complex excitation for the *k*th element at the *m-*th order harmonic frequency is given by

4 $$\begin{array}{rcl}{a}_{mk} & = & \frac{1}{{T}_{p}}{\int }_{0}^{{T}_{p}}{U}_{k}(t){e}^{-j2\pi {f}_{p}t}\cdot dt\\  & = & {\tau }_{k}{\rm{sinc}}(\pi m{\tau }_{k}){e}^{-j\pi m(2{t}_{k}+{\tau }_{k})}-(1-{\tau }_{k}){\rm{sinc}}(\pi m(1-{\tau }_{k})){e}^{-j\pi m(2{t}_{k}+{\tau }_{k}+1)}\\  & = & [({\tau }_{k}{\rm{sinc}}(\pi m{\tau }_{k})-{(-1)}^{m}(1-{\tau }_{k}){\rm{sinc}}(\pi m(1-{\tau }_{k}))){e}^{-j\pi m{\tau }_{k}}]{e}^{-jm(2\pi {t}_{k})}\end{array}$$

Similarly with the SPST switch, *a*_*mk*_ of the SPDT switch will also equal to the rotating factor of the OAM-carrying waves by setting *τ*_*k*_ to be the same and *t*_*k*_ = (*k* − 1)/*N* (*k* = 1, 2 …, *N*).

### Time sequence optimization for SPST switches and SPDT switches

**Figure 1:**
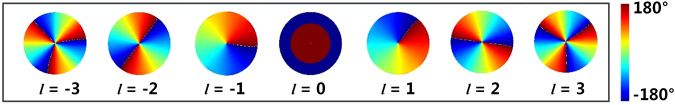
Phase images of OAM modes. Each mode is labelled by its topological charge *l*. A change in color from red to blue and back to red again corresponds to a change in phase of 360°.

**Figure 2:**
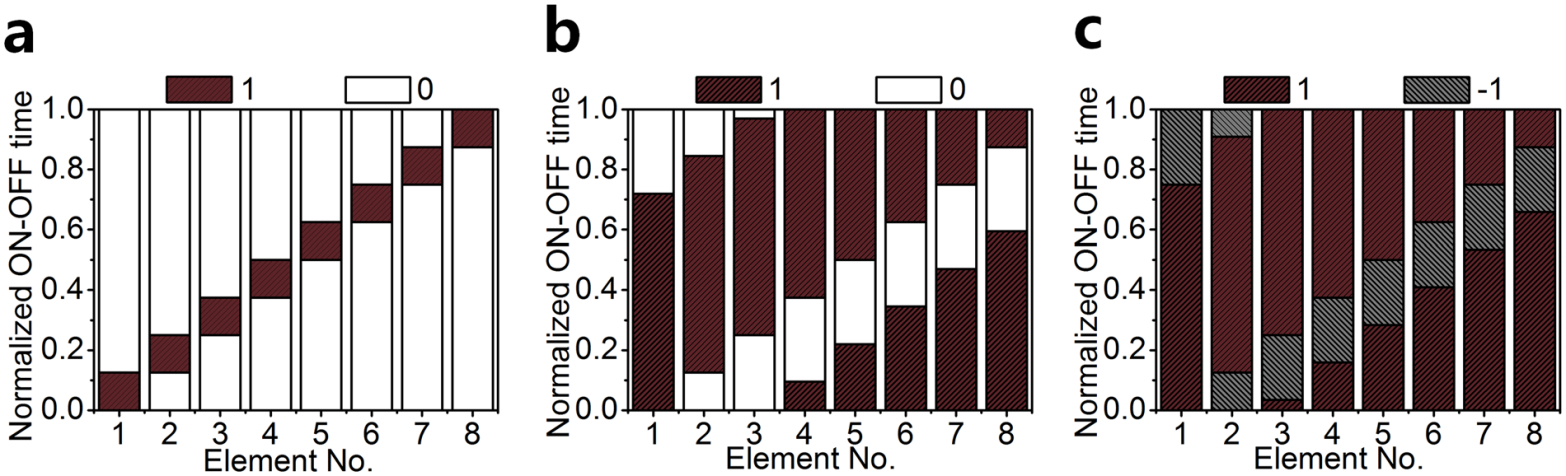
Time sequences distribution of the 8-element uniformly excited 4-D antenna array. (**a**) Proposed time sequence in ref.^10^ by using SPST switch. (**b**) Optimized time sequence by using SPST switch. (**c**) Optimized sequence by using SPDT switch. Red block represents the switch connects to “1” state, white block represents the switch connects to “0” state and gray block represents the switch connects to “−1” state.

**Figure 3:**
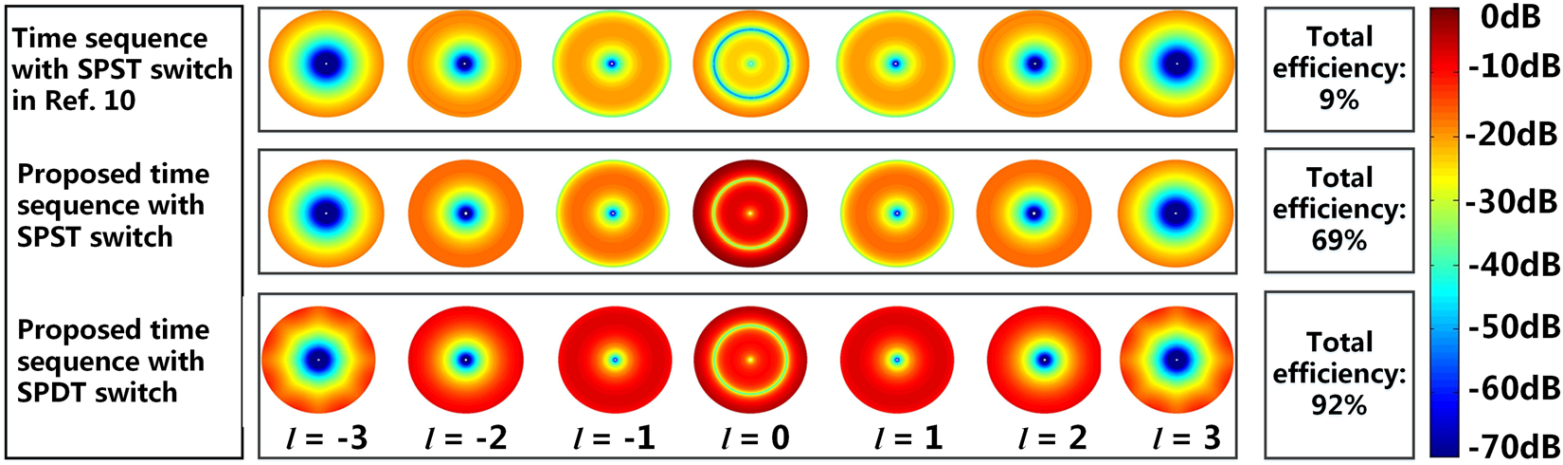
Amplitude images of OAM modes. Three different time sequence schemes are presented, including the time sequence with SPST switch in ref.^10^, the proposed time sequence with SPST switch and the proposed time sequence with SPDT switch. The simulated data is normalized to the maximum value. Each mode is labelled by its topological charge *l*. A change in color from blue to red corresponds to a change in amplitude from −70 dB to 0 dB.

In this simulation, an equidistantly spaced 8-element 4-D circular antenna array (*R* = 0.75*λ, λ* is the free-space wavelength at the operating frequency) of uniformly excited isotropic elements is considered. Under the aforementioned constraint of time sequence, the phase image of each OAM mode is shown in Fig. 1. As can be seen, the generated multiple OAM-carrying waves by 4-D circular antenna array possess both positive mode and negative mode, rotating in the opposite direction. For the *l-*th (*l* ≠ 0) mode generated at harmonic frequency, the twisted vortex phase front corresponds to a phase pattern with 2π•*l* phase rotation in one geometrical rotation. For the 0th (*l* = 0) mode generated at the center frequency, the phase fronts has the typical characteristic for plane or spherical wave. In ref.^10^, a time sequence as shown in Fig. 2a was proposed to generate OAM-carrying waves using SPST switch. The amplitude distributions of the OAM-carrying waves at ± *l-*th (*l* ≠ 0) order are symmetrical as shown in Fig. 3. Doughnut-shaped beam profile implies a null of power in the propagation direction. Owing to the short “switch-on” time duration, the amplitude of each mode is very low. Moreover, with the increasing of *l*, the weak-field area expands, leading to a shorter transmitting range. In order to improve the radiation power of each mode and the radiation efficiency of the 4-D circular antenna array with SPST switches, classical differential evolution algorithm is chosen as the global optimization method^20^. The optimized time sequence of the SPST switches is shown in Fig. 2b and the amplitude distributions of OAM-carrying wave is shown in Fig. 3. Compared with the time sequence in ref.^10^, the optimized time sequence with SPST switches in this work provides a significant enhancement on the amplitude of the 0th OAM mode. Meanwhile, the total efficiency of the 4-D circular antenna array with the optimized time sequence with SPST switches is 69%, which is much higher than the 9% efficiency in ref.^10^ (see Supplementary Note 2 for the time sequence optimization of SPST switches).

As for the SPDT switches^21,22^, the optimized time sequence is shown in Fig. 2c and the amplitude distributions of OAM-carrying wave are shown in Fig. 3. The proposed time sequence with SPDT switches greatly improves the amplitude of each OAM mode while minimizes the amplitude difference among the modes. The total efficiency of 4-D circular antenna array by using SPDT switches is 92%, which is significantly higher than using SPST switches (see Supplementary Note 2 for the time sequence optimization of SPDT switches). Therefore, it can be concluded that, the proposed time sequence with SPDT switches are more attractive in the simultaneous generation of multiple OAM modes.

### Experiment verification

In the experiment, an 8-element 4-D circular antenna array is designed to verify the above described approach of simultaneously generating multiple OAM modes. The operation frequency, the time modulated frequency, and the time modulated period are *f*_0_ = 2.6 GHz, *f*_*p*_ = 0.1 MHz, and *T*_*p*_ = 10000 ns, respectively. The radius of the designed 4-D circular antenna array is 0.75*λ*. The photograph of the detailed information of the antenna element design, the fabricated circular antenna array prototype and the photograph of the switching circuit are provided in Supplementary Note 3 and Supplementary Note 4.

**Figure 4:**
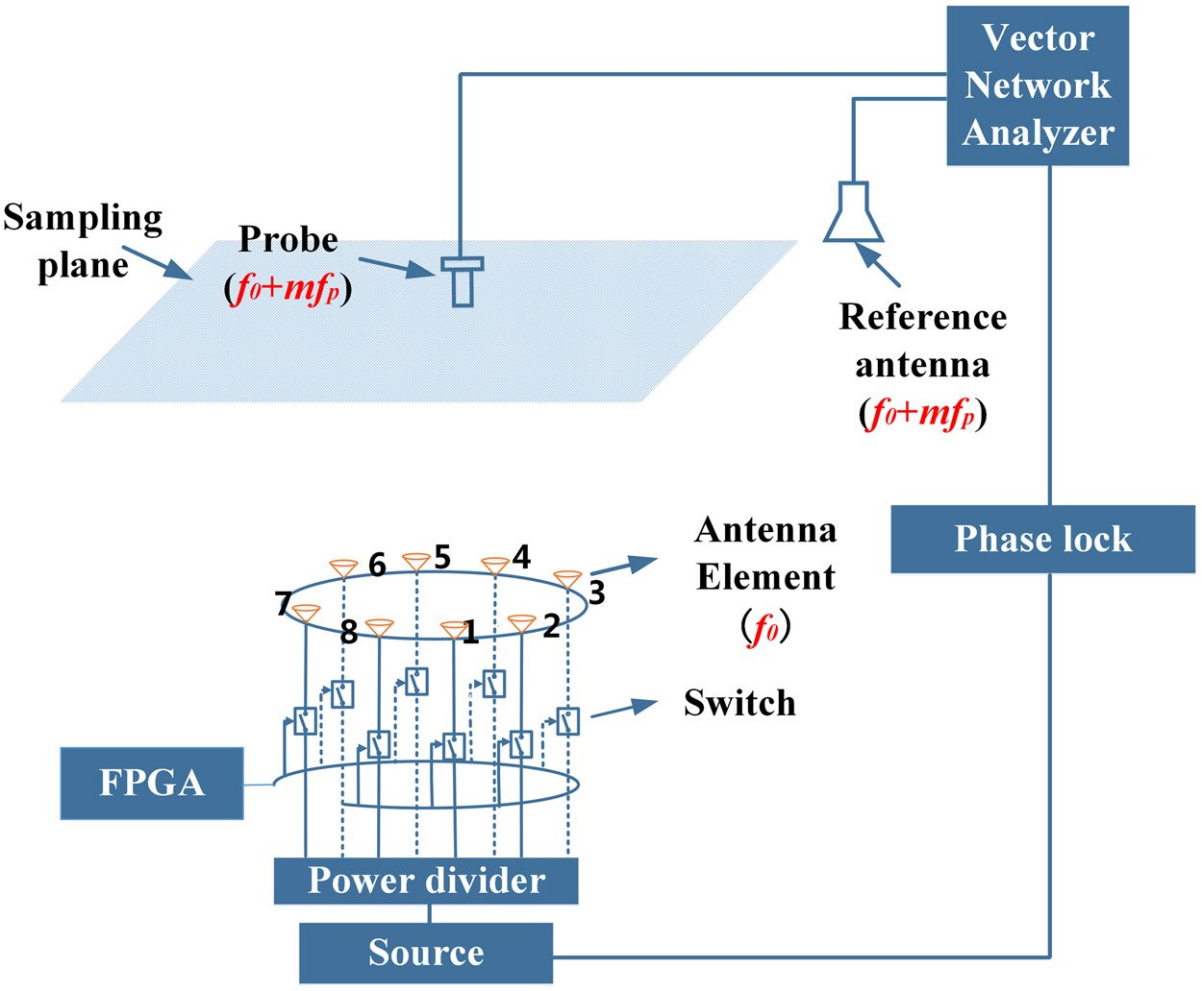
Near-field phase distribution measurement for 4-D antenna array. The transmitting antenna and the receiving antennas can operate at different frequencies. On the transmitting side, 4-D circular antenna array operates at center frequency *f*_0_, connecting with a function signal generator. On the receiving side, both the two receivers operate at the same frequency (harmonic frequency *f*_0_ ± *mf*_*p*_ or center frequency *f*_0_). One port of the vector network analyzer connects with a reference antenna to provide reference phase. The other port of the vector network analyzer connects with an open-ended rectangular waveguide that is used as a near-field probe. The measuring plane is 200 mm away from the array plane with a scan range of 500 mm × 500 mm.

For the characterization of the generated OAM-carrying waves, the phase distribution is of great importance. Generally, the near-field phase distribution of an antenna or an antenna array can be obtained with the traditional experimental platform in an anechoic chamber, where the transmitting antenna and the probe antenna operate at the same frequency. However, the simultaneously generated OAM-carrying waves by 4-D circular antenna array are distributed at multiple harmonic frequencies, other than only at the center frequency. As a result, the traditional experimental platform is not applicable for our measurement. To solve this issue, a novel experimental platform is developed in this paper and is shown in Fig. 4 (see Supplementary Note 5 for the photograph of the experiment setup in the anechoic chamber). As can be seen, a power divider is used to split the power from the source into eight ways with equal powers. A switching circuit is applied between the circular antenna array and the 1 × 8 power divider. A circuit board (FPGA) is used to control the switching circuit and thus the preset time sequence can be employed. In the experiment, the time sequence in Fig. 2b is adopted to generate the OAM-carrying waves.

In the experiment, the sampling plane is 200 mm above the circular antenna array with a scan range of 500 mm × 500 mm. To obtain the near-field distribution, an open-ended rectangular waveguide is used as the probe. As mentioned above, the key issue here is to address the difficulty in the measurement of the phase distribution. Because the phase of fields is a relative value instead of an absolute value, a reference antenna is added to provide a reference phase. The reference antenna is fixed while the probe antenna is scanning in the sampling plane. Meanwhile, both of the receiving ends of the open-ended rectangular waveguide probe and the reference antenna are connected to a two-port vector network analyzer (VNA). The VNA performs the function of comparing the two signals (see Supplementary Note 5 for details) from the open-ended rectangular waveguide probe and the reference antenna. Hence, the near-field phase distribution can be directly extracted from the measured ∠*S*_*21*_ from the VNA. Moreover, a phase locked loop is used to ensure that the vector network analyzer and the signal source are synchronized. To the author’s best knowledge, this issue has never been addressed before.

**Figure 5:**
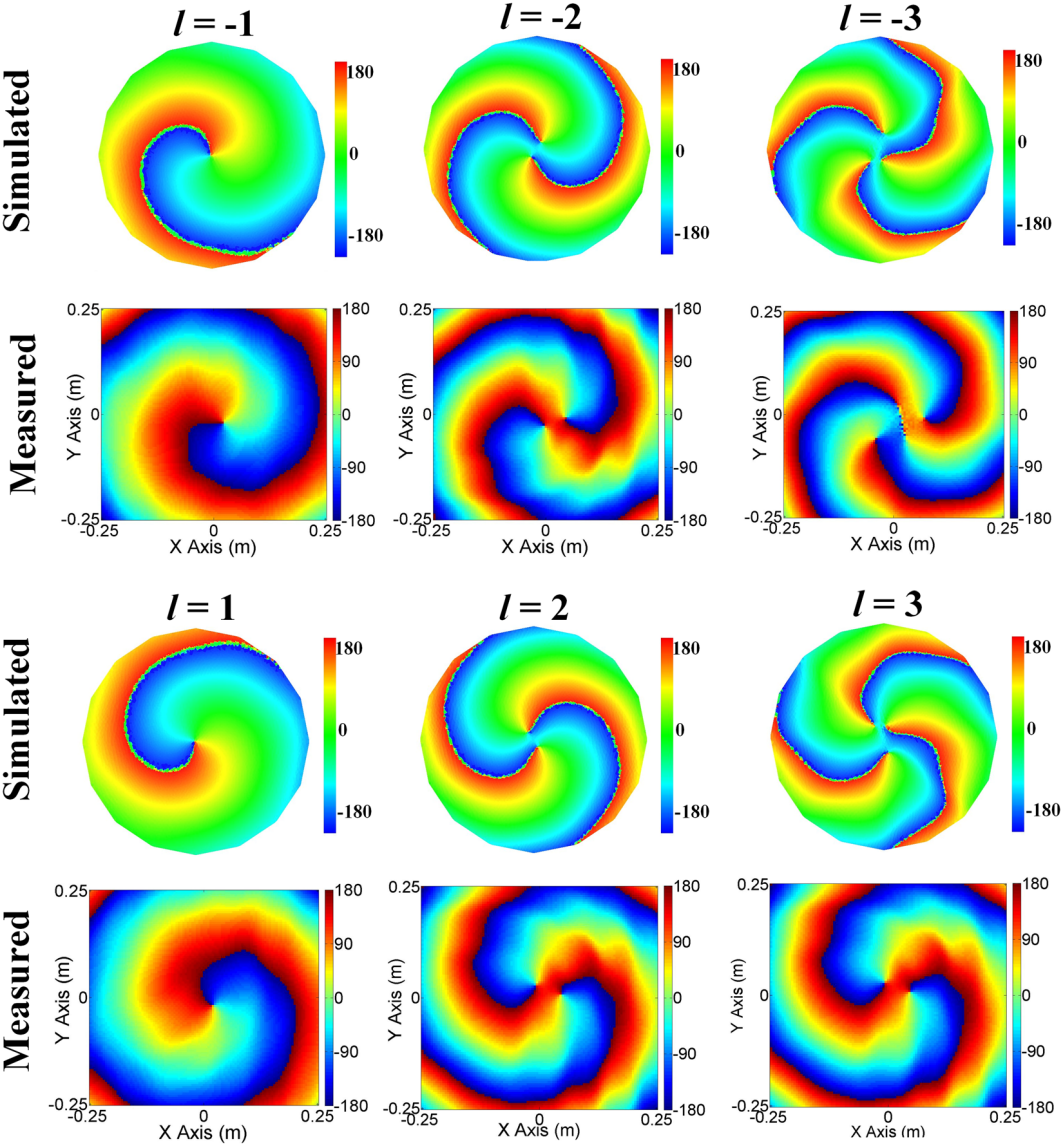
Simulated and measured Near-field phase distributions of OAM modes generated by 4-D antenna array. Each mode is labelled by its topological charge *l*. A change in color from red to blue and back to red again corresponds to a change in phase of 360°.

The simulated and the measured near-field distributions are shown in Fig. 5. In the measurement, 10201 sample spots are measured (scanning step: 5 mm). Detailed information of the simulation can be found in Supplementary Note 6. As seen from Fig. 5, the characteristic vortex phase fronts clearly indicate that OAM modes with *l* = ±1, *l* = ±2 and *l* = ±3 are simultaneous generated. The variation in color of the phase distribution of the generated *l-*th mode at the harmonic frequency corresponds to a phase change of 2π•*l* (*l* ≠ 0), respectively. It is obvious that the phase distributions of the positive modes (*l* = 1, *l* = 2 and *l* = 3) feature a clockwise increasing and that of the negative modes (*l* = −1, *l* = −2 and *l* = −3) feature an anticlockwise increasing. The high consistency of the measurement results, the simulated results and the theoretical results well demonstrates that 4-D circular antenna array is capable of simultaneously generating multiple OAM-carrying waves. The Measured near-field amplitude distributions of OAM modes generated by 4-D circular antenna array are shown in Supplementary Fig. S9.

### Demodulation of OAM-carrying signals

**Figure 6:**
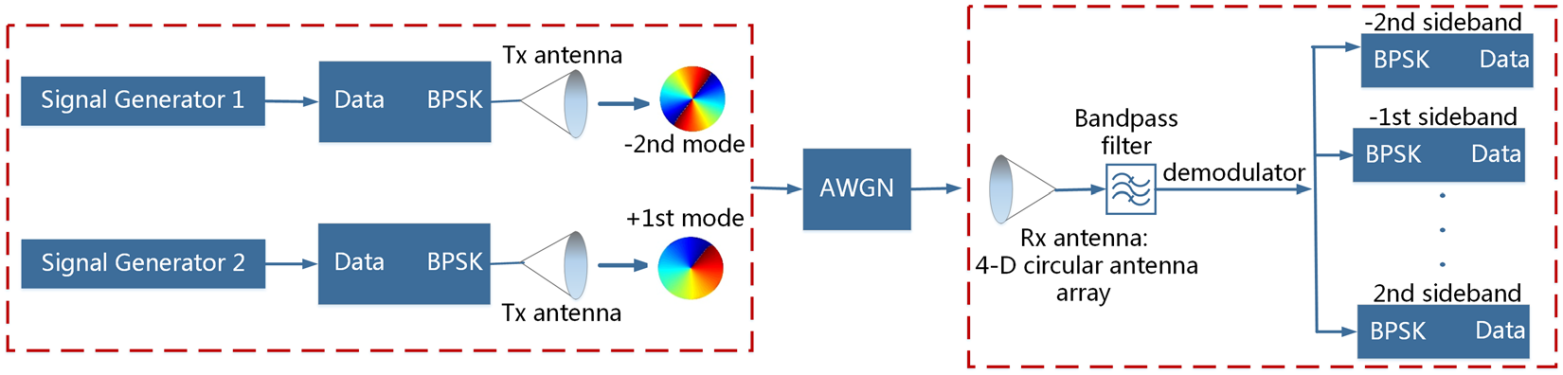
Scenario of demodulating the OAM-carrying signals. On the transmitting side, there are two binary phase shift keying (BPSK) signals carrying the −2nd OAM mode and the +1st OAM mode respectively. The signals operate at the frequency of 2.6 GHz and have a bit rate of *Rb* = 16 Mbps. The OAM-carrying BPSK signals are received and modulated by a 4-D circular antenna array, and a digital band-pass filter with a bandwidth of *B* = 0.5*f*_*p*_ is used to demodulate the BPSK signal. The power of the additive white Gaussian noise (AWGN) for different directions is set to be the same, while the power of the received signal corresponds to the radiation pattern.

**Figure 7:**
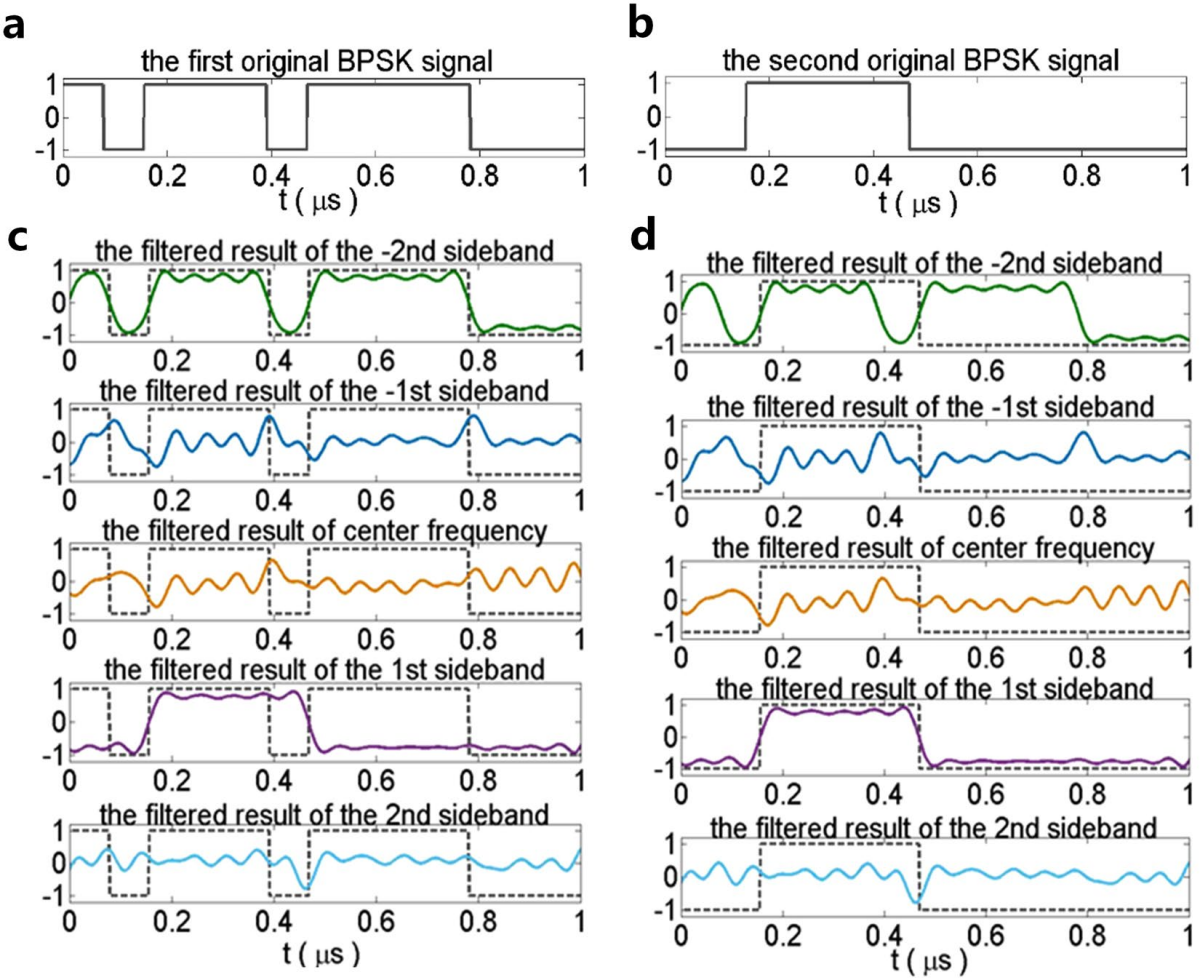
Simulated OAM-carrying BPSK signals waveform. (**a**) The first original BPSK signal. (**b**) The second original BPSK signal. (**c**) The demodulated signal at the center frequency and each harmonic frequency (solid line) and the first input original BPSK signal (dash line). (**d**) The demodulated signal at the center frequency and each harmonic frequency (solid line) and the second input original BPSK signal (dash line).

Due to the twisted phase front of OAM mode, OAM-carrying signal can only be recovered by a fixed antenna/antenna array structure. Meanwhile, the receiving antenna/antenna array is expected to generate the same OAM mode as the signal carrying. When considering multiple OAM-carrying signals coexist in free space (or even the mode values are uncertain), massive types of antenna/antenna array are needed on the receiving side to recover the original signals. This stems from the limitation that most of the existing antenna/antenna array structures can only produce a single mode at an instant. However, a 4-D circular antenna array with enough element number can recover all the signals simultaneously at corresponding harmonic frequencies. It also alleviates the burden on hardware implementations and cost. Figure 6 shows the scenario of demodulating the OAM-carrying signals by 4-D circular antenna array. On the transmitting side, there are two binary phase shift keying (BPSK) signals carrying the -2nd OAM mode and the +1st OAM mode respectively. The signals operate at the frequency of 2.6 GHz and have a bit rate of *Rb* = 16 Mbps, received and modulated by a 4-D circular antenna array. In multi-beam 4-D array, the center frequency and harmonic signals are usually separated from each other to avoid aliasing effects (overlapping of the modulated signal spectrum) (see Supplementary Note 8).Thus, a digital band-pass filter with a bandwidth of *B* = 0.5*f*_*p*_ is used to demodulate the OAM-carrying BPSK signals. The power of the additive white Gaussian noise (AWGN) for different directions is set to be the same, while the power of the received signal corresponds to the radiation pattern. Figure 7 shows the simulated waveforms (replicated into two columns) of the OAM-carrying BPSK signals that are filtered at center frequency and harmonic frequencies, compared with the two original BPSK signals respectively. The solid line in Fig. 7a and the dash lines in Fig. 7c illustrate the waveforms of the first original BPSK signal. Similarly, the solid line in Fig. 7b and the dash lines in Fig. 7d illustrate the waveforms of the second original BPSK signal. Figure 7c illustrates the comparison waveforms of the demodulated signal (solid lines) and the first original signal (dash lines). As expected, the first original BPSK signal (carrying the −2nd mode) can be fully recovered at the −2nd sideband, while the waveforms of the signals filtered at center frequency and other harmonic frequencies are changed. Similarly, the second BPSK signal (carrying the +1st mode) can only be recovered at the +1st sideband as shown in Fig. 7d. Therefore, 4-D circular antenna array is beneficial to receive and recover multiple OAM-carrying signals simultaneously with a filter at the corresponding harmonic frequencies. (see Supplementary Note 9 for the further investigation).

## Discussion

Measurement results have been presented to demonstrate that a 4-D circular antenna array can be configured to generate multiple OAM-carrying waves simultaneously. As for the phase measurement, the proposed experimental platform is applicable for the scenario in which the transmitting and receiving antenna operate at different frequencies. At the same time, optimal time sequences of both SPST switches and SPDT switches are obtained for the generation of OAM-carrying waves. The simulation of filtering and BER (see Supplementary Note 9) further validate the advantages of 4-D circular antenna array in demodulating the OAM-carrying signals simultaneously and simplifying the hardware implementation. All of these attractive features illustrate that the proposed 4-D circular antenna array is a promising candidate for radio communications and radar applications.

## Methods

### Simulation

All numerical results in the main text are calculated by MATLAB, including the optimization of time sequence and the demodulation of OAM-carrying signals. The simulated results of four-dimensional (4-D) circular antenna array are obtained by High Frequency Structure Simulator (HFSS) which is based on Finite Element Method (FEM).

### Experimental setup

In the experiment, measurement is carried out with a novel experimental platform in the anechoic chamber. An Agilent N9310A RF Signal Generator and an Agilent PNA-X N5242A Network Analyzer are employed. The Agilent PNA-X 5242A Network Analyzer is used to measure near-field phase distribution of the generated multiple OAM modes.

## Electronic supplementary material

Supplementary Information
