# Supplementary material for: Realization of multiple orbital angular momentum modes simultaneously through four-dimensional antenna arrays
Source: Sci Rep. 2018 Jan 9;8:149. doi: 10.1038/s41598-017-18264-3 (PMC5760727; doi:10.1038/s41598-017-18264-3)
Supplement: Supplementary file 1 — Supplementary Information [file 41598_2017_18264_MOESM1_ESM.doc]

# Realization of multiple orbital angular momentum modes simultaneously through four-dimensional antenna arrays

Chao Sun, Shiwen Yang*, Yikai Chen, Jixin Guo & Shiwei Qu

School of Electronic Engineering, University of Electronic Science and Technology of China (UESTC), 2006Xiyuan Avenue, Western High-Tech District, Chengdu 611731, China.

Corresponding author: Shiwen Yang

Email: swnyang@ uestc.edu.cn

Phone: +86-28-61830667

Chao Sun: 1551454624@qq.com

Yikai Chen: ykchen@ uestc.edu.cn

Jixin Guo: [809987212@qq.com](mailto:809987212@qq.com)

Shiwei Qu:  [shiweiqu@uestc.edu.cn](mailto:shiweiqu@uestc.edu.cn)

**Supplementary Note 1. Configuration of the 4-D Circular Antenna Array**


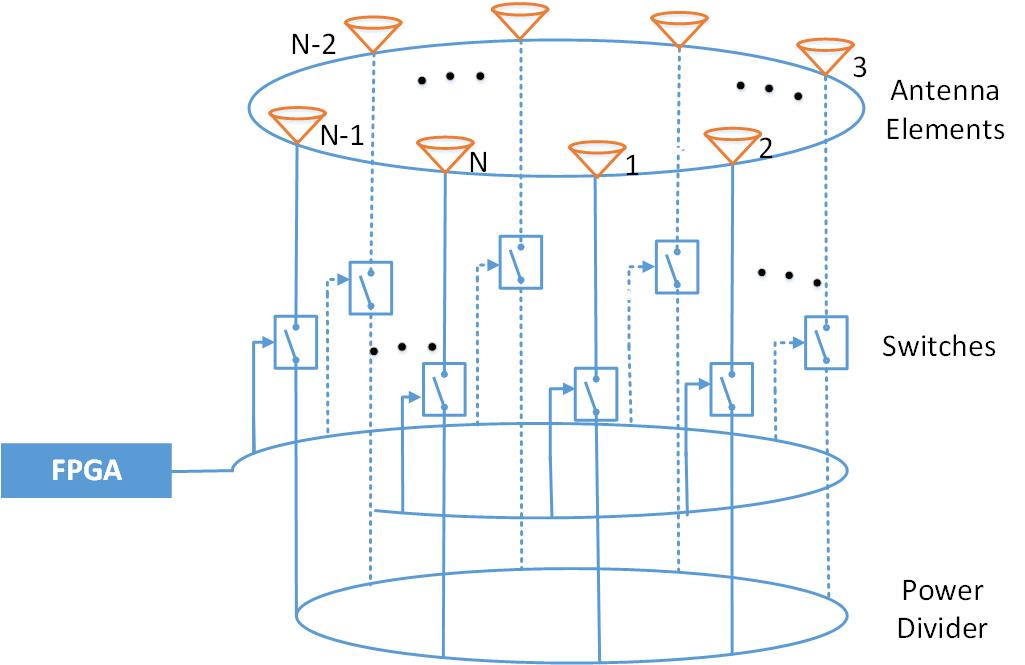


**Figure S1.** **Configuration of the 4-D circular antenna array for generating OAM-carrying waves.** An *N*-element 4-D circular antenna array with its elements placed equidistantly along a circle with a radius of *R*. Each antenna element is connected to a high speed RF switch, which is controlled by the circuit board (FPGA) for specific time sequences. The RF input signal is fed to the *N* switches through a 1-to-*N* way power divider.

**Supplementary Note 2. Time Sequence Optimization**

In order to improve the radiation power of each OAM mode and the radiation efficiency of the 4-D circular antenna array with SPST switches, the optimization parameter vector is formed as *v* = {*τ_k_*}. The power of each generated OAM mode and the total efficiency of the generated OAM modes constitute the cost function. The cost function is constructed as follows

$f\left(v\right)=\sum_{m=0}^{L} w_{m}\cdot P_{m}\left(v\right)\left|_{f_{0}+mf_{p}}\right.+w_{L+1}\cdot\left(\eta_{d}-\eta\left(v\right)\right)$ (1)

where *P_m_*, *m* ϵ [0 : *L*] (*L* = *N*/2-1, *N* is even; or *L* = (*N*-1)/2, *N* is odd) denotes the simulated radiation power of *m-*th OAM mode, respectively. Due to the symmetry of the ±*m-*th mode in radiation power, the cost function in Equation (1) takes the radiation power of the 0th mode and positive modes into consideration. *P_m_* is given by

(2)

*η* and *η_d_* = 1 are the simulated and desired total efficiency of the OAM-carrying waves generated by the 4-D circular antenna array. The total efficiency is defined as the ratio of the sum of the powers of all the generated OAM modes to the total power fed into the 4-D circular antenna array, and is given by

(3)

where *P_OAM_* represents the total power of the generated OAM modes, while *P_total_* and *P_r_* are the total power fed into the 4-D circular antenna array and the total radiation power of the 4-D circular antenna array, respectively. *η_f_* ($\eta_{_{f}}=\sum_{k=1}^{N} \tau_{k}/N$) represents the ideal efficiency of the feed network by using SPST switch, where actual power loss is not considered. *w_m_* (*m* ϵ [0 : *L*]) and *w_L_*_+1_ are the corresponding weighting factors of each term, which are given by *w*_0_ = 1,*w*_1_ = *w*_2_ = *w*_3_ = 2 and *w*_4_ = 100 in this work. Classical differential evolution (DE) algorithm is chosen as the global optimization method. Intrinsic parameters in classical DE are set as follows: *N_PAR_* is the optimization parameters, the population size *N_POP_* = 5 *N_PAR_*, the mutation probability *β* = 0.6, and the crossover probability *p_cross_* = 0.9. The optimized time sequence with SPST switch and the amplitude of each OAM-carrying wave are shown in Fig. 2b and Fig. 3 in the main text. As compared with the time sequence in Ref. 10 in the main text, the optimized time sequence with SPST switches in this work provides a significant enhancement on the amplitude of the 0th OAM mode. Meanwhile, the total efficiency of the 4-D circular antenna array with the optimized time sequence using SPST switches is 69%, which is much higher than the 9% efficiency in Ref. 10 in the main text.

As for the SPDT switches, the optimization parameter vector and the cost function are the same as the SPST switches, except for the feed network efficiency *η_f_*. Due to the topology of the SPDT, the switches are theoretically always in the “ON” state and there is no energy wasted in the feed network. Therefore, we apply *η_f_* = 1 in Equation (3). The optimized time sequence with SPDT switch and the amplitude of each OAM-carrying wave are shown in Fig. 2c and Fig. 3 in the main text. The proposed time sequence with SPDT switches greatly improves the amplitude of each OAM mode while minimizes the amplitude difference among the modes. The total efficiency of 4-D circular antenna array by using SPDT switches is 92%, which is significantly higher than using SPST switches. Therefore, it can be concluded that, the proposed time sequence with SPDT switches are more attractive in the simultaneous generation of multiple OAM modes.

**Supplementary Note 3. Antenna Element Design**

The structure of the proposed E-shaped patch element is presented in Fig. S2a. The antenna is printed on a 3.175 mm thick Taconic TLX substrate which has a relative permittivity of 2.5 and a loss tangent of 0.0019. Fig. S2b shows the reflection coefficient (*S*_11_) of a single E-shaped patch antenna. As can be seen from Fig. S2b, the patch antenna has -10 dB *S*_11_ bandwidth from 2.48 GHz to 2.84 GHz.


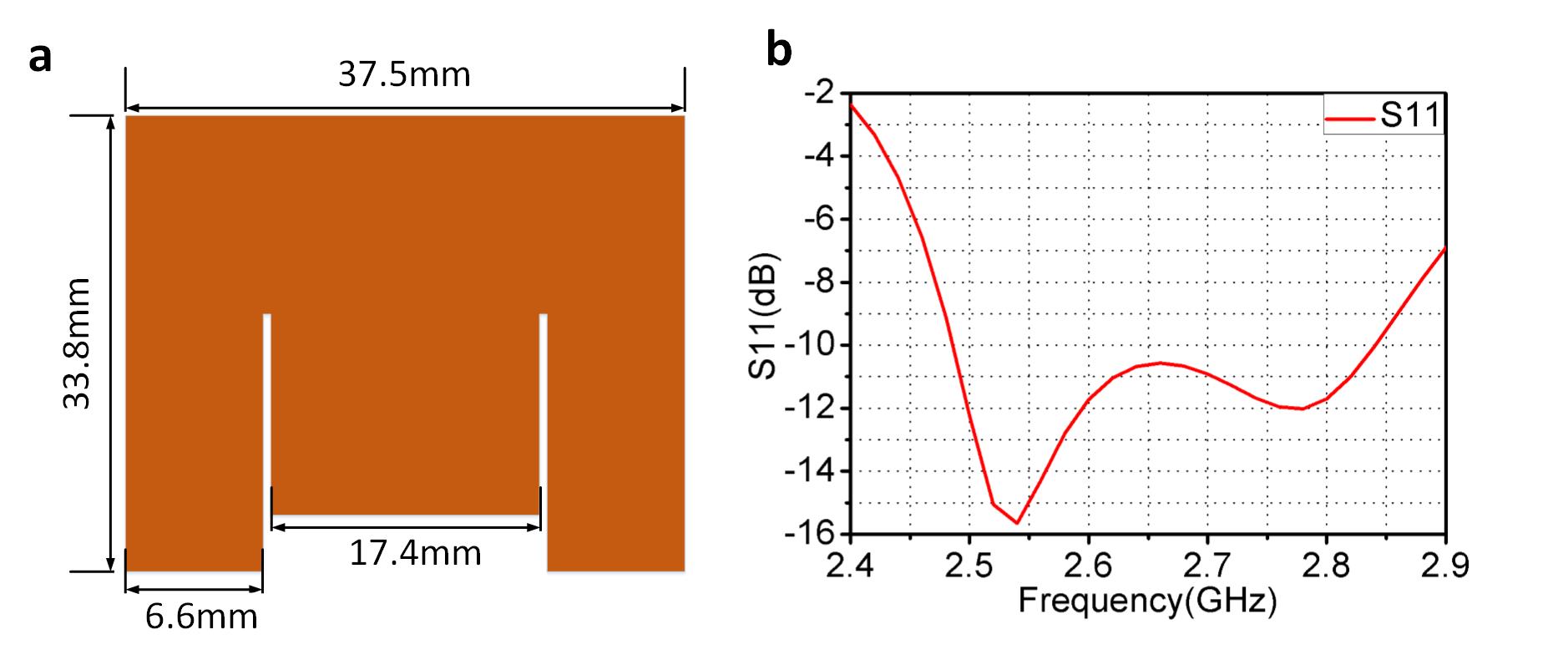


**Figure S2.** **Single E-shaped patch element**. (**a**) Structure. (**b**) Reflection coefficient (*S*_11_).

**Supplementary Note 4. Prototype of the 4-D Circular Antenna Array and the Feed Network**

Fig. S3a shows the geometry of the proposed 8-element circular antenna array with a radius of 0.75*λ* which is designed based on the E-shaped patch element. The feed network of the four-dimensional (4-D) circular antenna array is shown in Fig. S3b. Each element is connected to a high speed RF switch and the switches are controlled by a circuit board (FPGA). The commercial RF switches in Fig. S3b are absorptive single-pole single-throw, with a high switching speed and a high isolation over a wide frequency range from 10 MHz to 4 GHz. It has a maximum switching speed of 6 ns and an insertion loss of 3dB. The operation frequency, time modulated frequency, and time modulated period of the 4-D circular antenna array are *f_0_* = 2.6 GHz, *f_p_* = 0.1 MHz, and *T_p_* = 10000 ns, respectively.


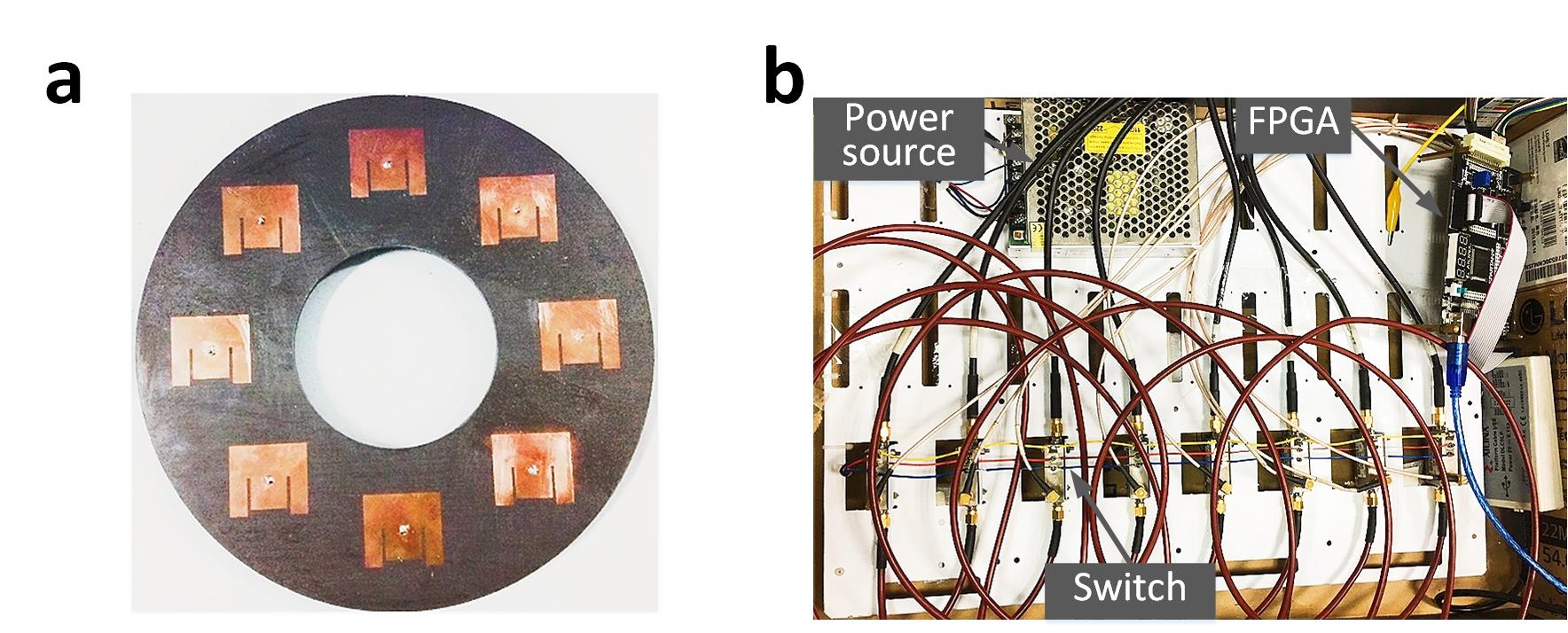


**Figure S3. Prototype of the 4-D circular antenna array.** (**a**) 8-element E-shaped patch circular antenna array. A Taconic TLX substrate with *ε*_r_ = 2.5 and a thickness *h* = 3.175 mm is used. (**b**) Feed network of the 4-D circular antenna array.

**Supplementary Note 5. Experimental Setup**

The experimental setup in the anechoic chamber for near-field phase distribution measurement of the proposed 4-D circular antenna array is shown in Fig. S4, which is set up based on Fig. 4 in the main text.

In our measurement, a special function of the VNA, called ‘arbitrary ratio’ function, is enabled. In this case, both of the two ports of the VNA are used as the receiving ports. With the build-in function of the VNA, we compare the signals from the two receiving ports when the above mentioned ‘arbitrary ratio’ function is enabled. The phase distribution thus can be directly extracted from the S parameter measured from the VNA. The description of the ‘arbitrary ratio’ function in the VNA is shown in Fig. S5.


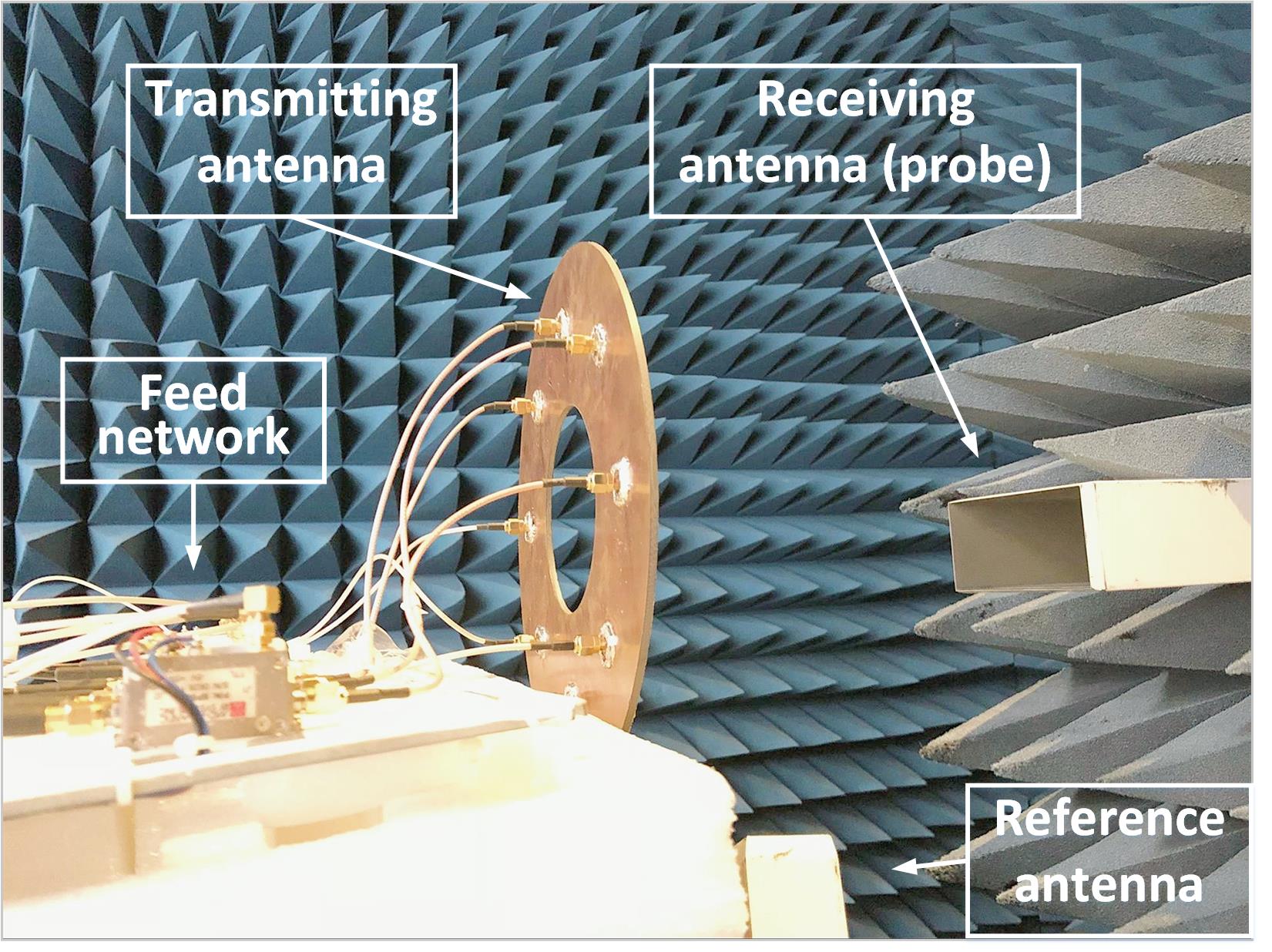


**Figure S4. Experimental setup for the near-field phase distribution measurement of the 4-D circular antenna array.**


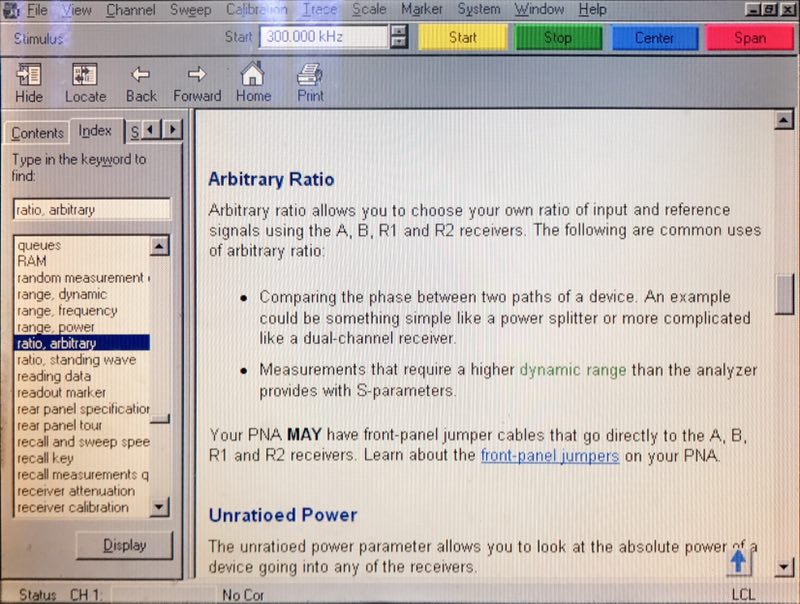


**Figure S5. Screenshot of the description of the ‘arbitrary ratio’ function in the VNA.**

**Supplementary Note 6. Simulation Verification**

The simulated results of the four-dimensional (4-D) circular antenna array are obtained by High Frequency Structure Simulator (HFSS) which is based on the Finite Element Method (FEM). The simulation model is shown in Fig. S6a. The excitation coefficients of the antenna elements are obtained by the Fourier transform of the optimized time sequence shown in Fig. 2b. The simulation model with a cut-plane 200 mm above the designed circular antenna array is shown in Fig. S6b. The cut-plane is in parallel to the *xoy* plane and has a radius of 250 mm. The simulated near-field phase distributions of the generated OAM modes are shown in Fig. S7. The characteristic vortex phase fronts in Fig. S7 clearly indicate that OAM modes with *l* = ±1, *l* = ±2 and *l* = ±3 are generated.

As a further and more intuitive way to distinguish the generated OAM mode order, the phase distributions along the circumference of a circle (radius: 100 mm) on the aforementioned cut-plane is extracted from the full-wave simulations and are plotted in Fig. S8. As can be seen from Fig. S8, the phase distributions are linear to the angular offset and the gradient of the phase distribution of each OAM mode approximately equals to its corresponding topological charge *l*^1,2^, respectively.


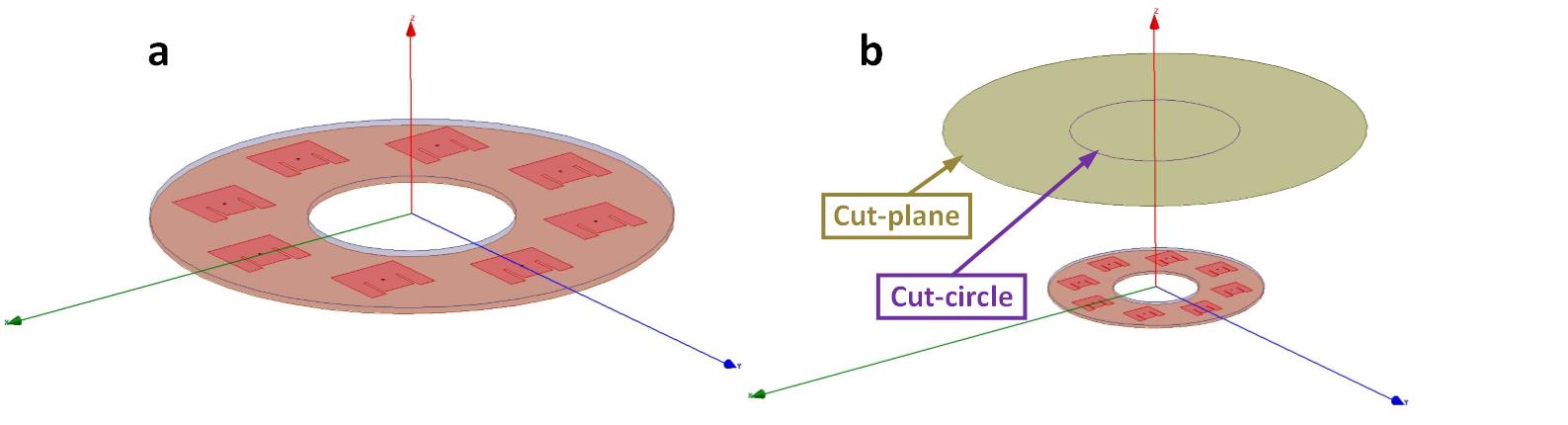


**Figure S6. Simulation model.** (**a**) Simulation model of the 4-D circular antenna array. (**b**) Simulation model of the 4-D circular antenna array with both the cut-plane and the cut-circle. The cut-plane with a radius of 250 mm is 200 mm above the designed circular antenna array and is in parallel to the *xoy* plane. The radius of the cut-circle on the cut-plane is 100 mm.


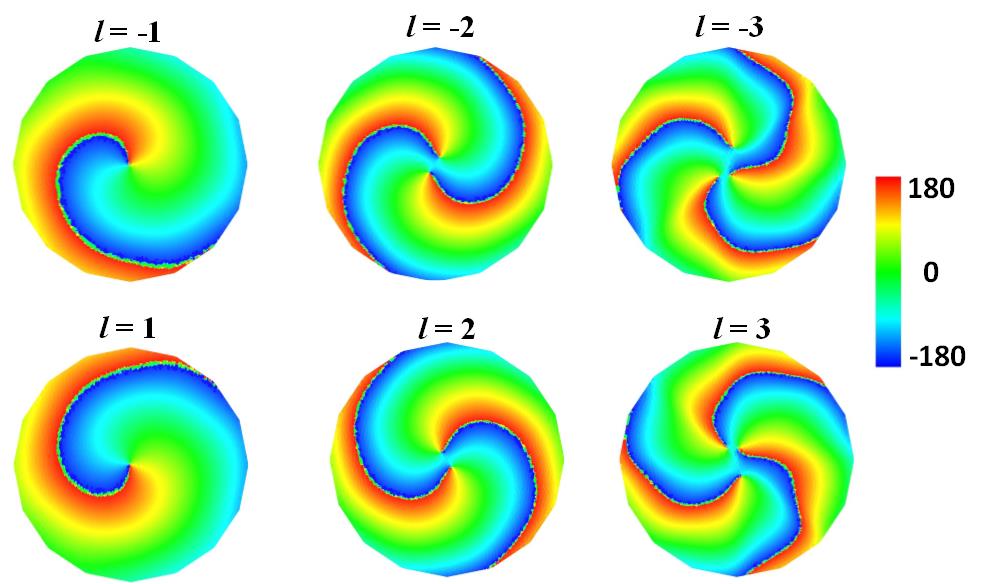


**Figure S7. Simulated near-field phase distributions of the OAM modes generated by 4-D circular antenna array.** Each mode is labelled by its topological charge *l*. A change in color from red to blue and back to red again corresponds to a change in phase of 360°.


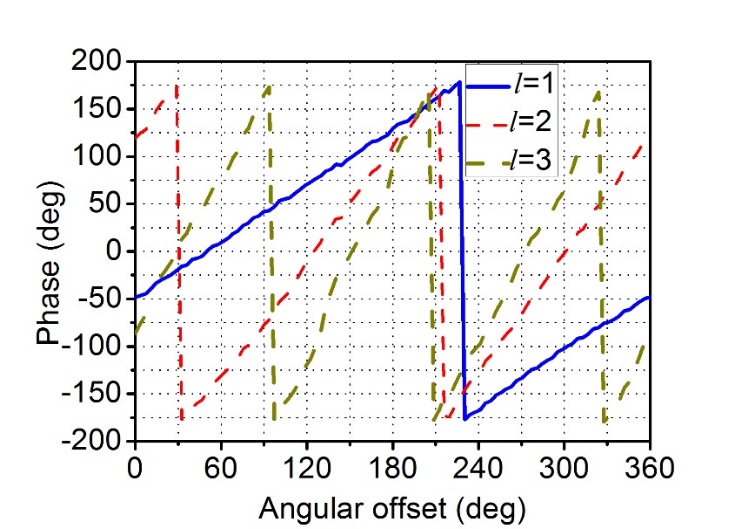


**Figure S8. Phase profiles of the generated OAM modes with *l* = 1, *l* = 2 and *l* = 3.**

**Supplementary Note 7. Measured Amplitude Distributions**

The measured near-field amplitude distributions of OAM modes generated by 4-D circular antenna array are shown in Fig. S9. The doughnut-shaped high-intensity profile is clearly seen, and the higher-order radiation modes correspond to larger on-axis null regions. The experiment results show a good agreement with the simulated results.


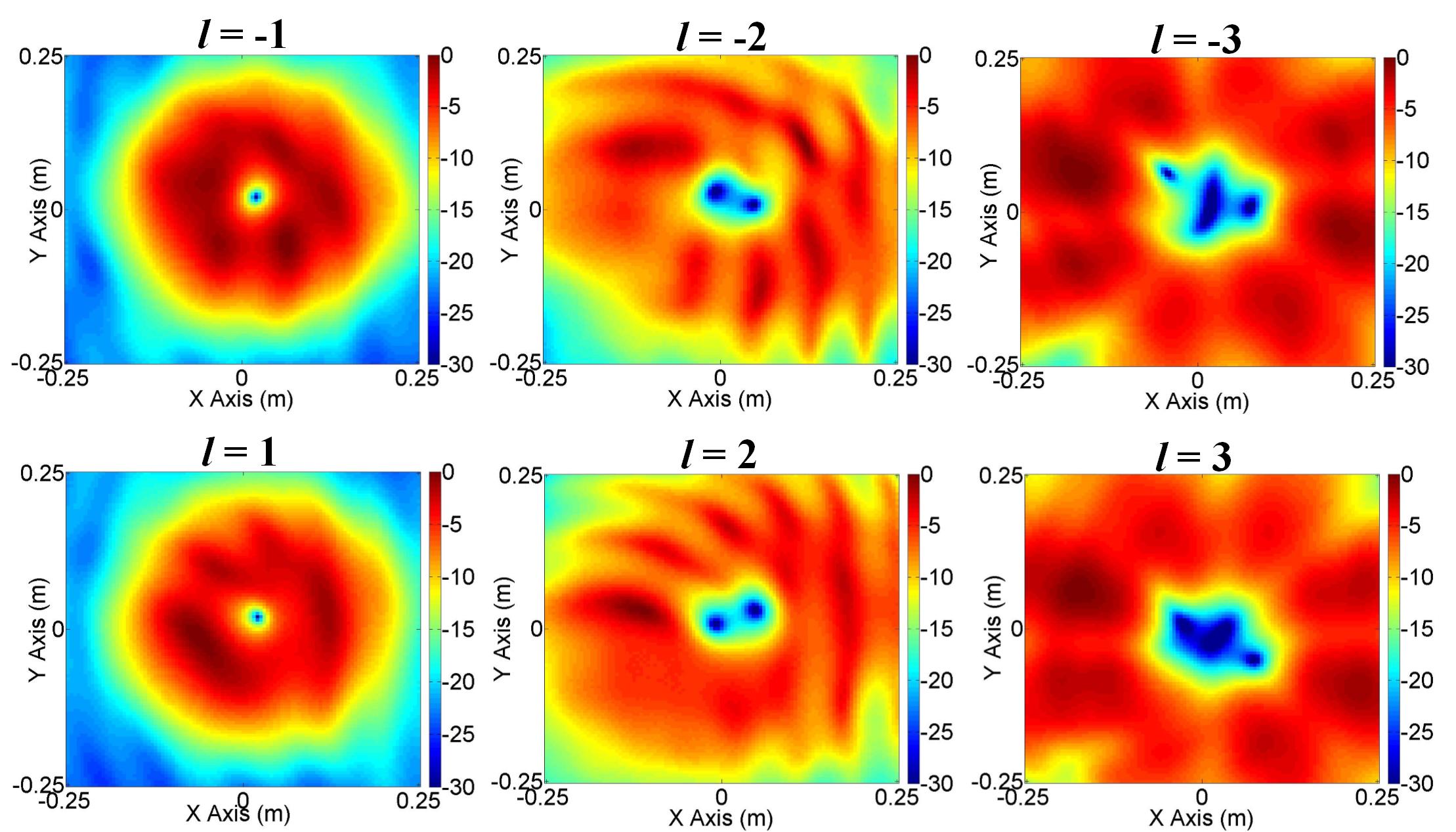


**Figure S9.** **Measured** **near-field amplitude distributions of OAM modes generated by the 4-D circular antenna array.** The measured data is normalized to the maximum value. Each mode is labelled by its topological charge *l*. A change in color from red to blue corresponds to a change in amplitude from 0 dB to -30 dB.

**Supplementary Note 8. Signal Spectrum**

In multi-beam 4-D antenna array, the center frequency and harmonic signals are usually separated from each other to avoid aliasing effects (overlapping of the modulated signal spectrum). The time modulation frequency *f_p_* must be greater than the signal bandwidth *B*, such that the original signal at the center frequency or harmonic signals can be recovered by using a band-pass filter (shown in Fig. S10). Thus, a digital band-pass filter with a bandwidth of *B* = 0.5*f_p_* is used to demodulate the OAM-carrying BPSK signals in the main text.


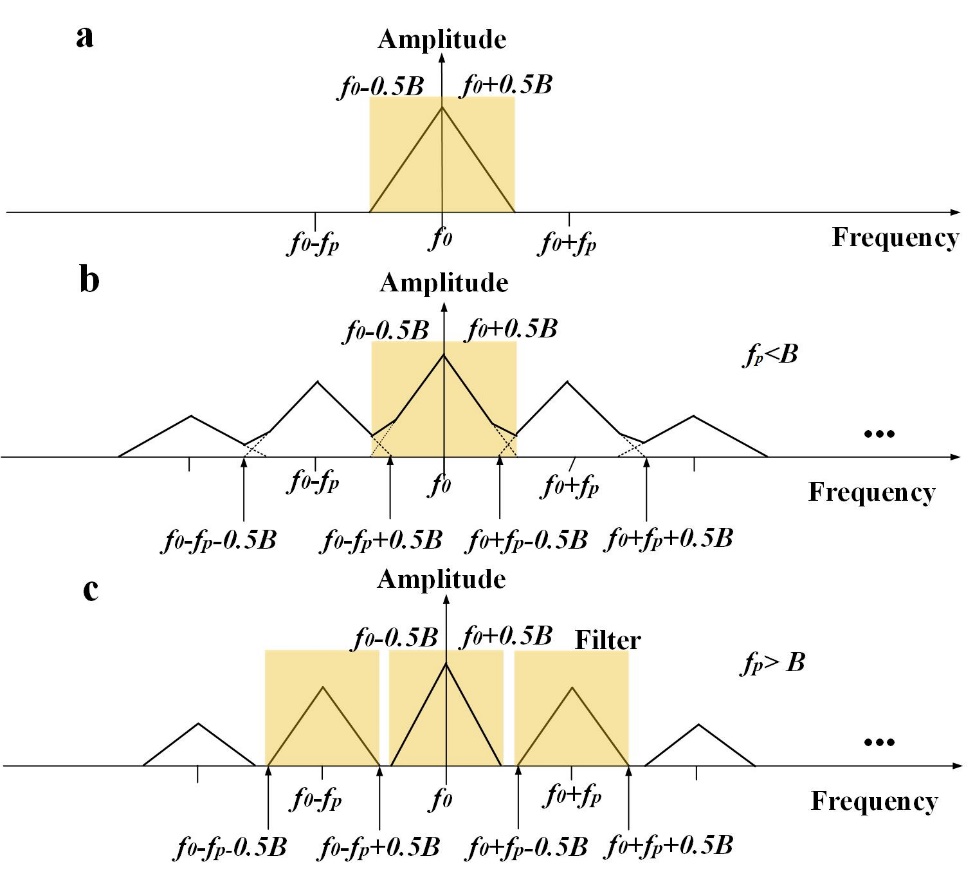


**Figure S10. Signal spectrum**. (**a**) Original input signal where no sideband signals exist. (**b**) Received signals where sideband signals exist (*f_p_* < *B*). (**c**) Received signals where sideband signals exist (*f_p_* > *B*).

**Supplementary Note 9. BER Performance versus SNR**

In the main text, the capability of 4-D circular antenna array in receiving and demodulating OAM-carrying BPSK signals are investigated through the filtered waveforms. To further investigate the proposed approach, bit error ratio (BER) performance of the signals received and demodulated by 4-D circular antenna array is studied. Monte Carlo approach is introduced in this simulation, since it is usually used to simulate the signal BER in communication systems. Fig. S11 shows the BER performance of the demodulated OAM-carrying BPSK signal versus signal-to-noise ratio (SNR) at center frequency and several harmonic frequencies. With the increase of the SNR, the BER of the first BPSK signal (carrying the -2nd mode) demodulated at the -2nd harmonic frequency decreases rapidly, while the BER at center frequency and other harmonic frequencies are very high (shown in Fig. S11a). Therefore, the BPSK signal carrying the -2nd mode can only be fully recovered at the -2nd sideband. Similarly, the BPSK signal carrying the +1st OAM mode can only be fully recovered at the +1st sideband (shown in Fig. S11b).


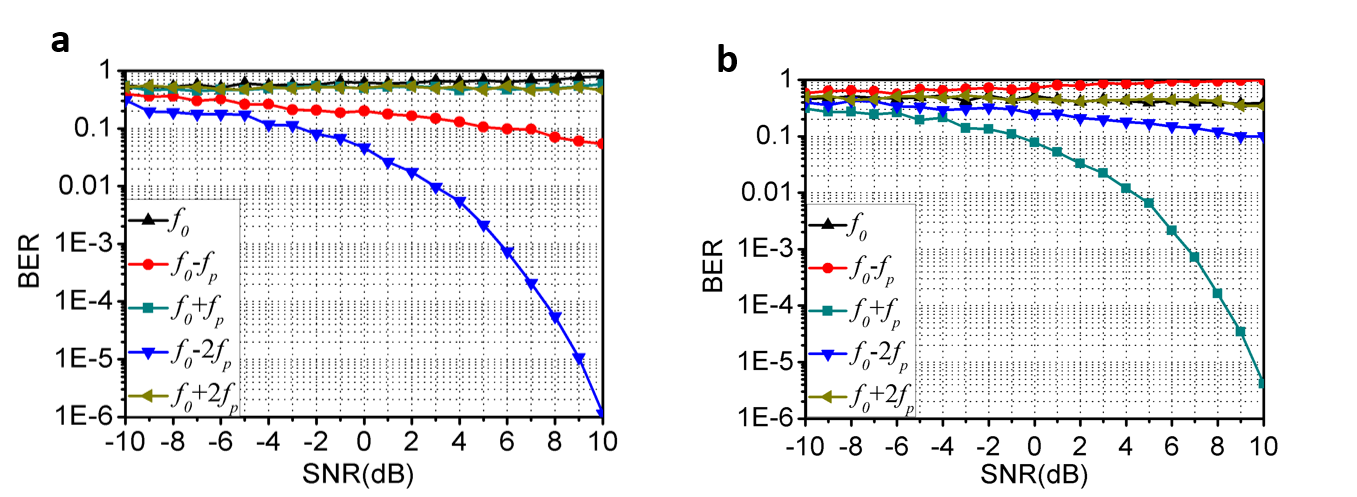


**Figure S11.** **BER performance versus SNR** **for the OAM-carrying BPSK signal received by the 4-D circular antenna array**. (**a**) BPSK signal carries the -2nd OAM mode. (b) BPSK signal carries the +1st OAM mode.

**Reference**

1. Kang, L. *et al*. Generation of OAM Beams Using Phased Array in the Microwave Band. *IEEE Tran. Antennas Propagat.* **64**, 3850-3857 (2016).
2. Mohammadi, S.M. *et al*. Orbital angular momentum in radio: measurement methods. *Radio Sci*.,**45**, 1–14 (2010).
